# Progressive Changes in Inflammatory and Matrix Adherence of Bronchial Epithelial Cells with Persistent Respiratory Syncytial Virus (RSV) Infection (Progressive Changes in RSV Infection)

**DOI:** 10.3390/ijms140918024

**Published:** 2013-09-03

**Authors:** Xiaoai Liu, Xiaoqun Qin, Yang Xiang, Huijun Liu, Ge Gao, Ling Qin, Chi Liu, Xiangping Qu

**Affiliations:** 1 Department of Physiology, School of Basic Medical Science, Central South University, Changsha 410078, China; E-Mails: alicelxa@gmail.com (X.L.); xiangyang@mail.csu.edu.cn (Y.X.); liuhj82913@gmail.com (H.L.); ggboxy13@gmail.com (G.G.); liuchi7669@gmail.com (C.L.); quxiangping@mail.csu.edu.cn (X.Q.); 2 Department of Physiology, Guangzhou Medical University, Guangzhou 510182, China; 3 Respiratory Department, Xiangya Hospital, Central South University, Changsha 410078, China; E-Mail: qlmelody13@gmail.com

**Keywords:** respiratory syncytial virus, human bronchial epithelial cells, adherence, adhesive molecule, cytokine, chemokine

## Abstract

In addition to the acute manifestations of respiratory syncytial virus (RSV), persistent infection may be associated with long-term complications in the development of chronic respiratory diseases. To understand the mechanisms underlying RSV-induced long-term consequences, we established an *in vitro* RSV (strain A2) infection model using human bronchial epithelial (16HBE) cells that persists over four generations and analyzed cell inflammation and matrix adherence. Cells infected with RSV at *multiplicity of infection* (*MOI*) 0.0067 experienced cytolytic or abortive infections in the second generation (G2) or G3 but mostly survived up to G4. Cell morphology, leukocyte and matrix adherence of the cells did not change in G1 or G2, but subsequently, leukocyte adherence and cytokine/chemokine secretion, partially mediated by intercellular adhesion molecule-1 (ICAM-1), increased drastically, and matrix adherence, partially mediated by E-cadherin, decreased until the cells died. Tumor necrosis factor-α (TNF-α) secretion was inhibited by ICAM-1 antibody in infected-16HBE cells, suggesting that positive feedback between TNF-α secretion and ICAM-1 expression may be significant in exacerbated inflammation. These data demonstrate the susceptibility of 16HBE cells to RSV and their capacity to produce long-term progressive RSV infection, which may contribute to inflammation mobilization and epithelial shedding.

## 1. Introduction

Respiratory syncytial virus (RSV) is a member of the family *Paramyxoviridae*, subfamily *Pneumovirinae*, which is the most common worldwide cause of epidemic respiratory diseases in children and bronchiolitis/pneumonia in infants, the elderly, and the immunocompromised [1,2]. The occurrence of RSV outbreak is related to unfavorable outcomes, including respiratory distress and death [3–7]. Consequently, RSV is an extremely expensive health problem for individuals, governments, and health care systems. Unfortunately, to date there are no commercially available vaccines against this pathogen and no effective post-infection treatments [8]. In addition to the acute manifestations of infection, RSV is thought to be associated with long-term complications, such as recurrent wheezing and asthma symptoms [9,10]. Recent implication of RSV persistence in the development of chronic respiratory diseases has renewed its interest [11]. Levels of persistent low-grade RSV infection may correlate with the incidence and severity of chronic obstructive pulmonary disease [12]. Because no animal reservoir has been described for this virus, its persistence in humans may explain not only these sequelae but also viral maintenance between seasonal epidemics. Furthermore, the persistence of RSV may be an underlying cause of recurrent outbreaks in long-term care facilities, neonatal intensive care units and pediatric intensive care units [3–7].

Bronchial epithelial cells are the site of RSV infection and replication. These cells play an important role in maintaining the normal homeostasis of the respiratory system by providing a physiological barrier against the external milieu of the airway through the formation of tight junctions. Additionally, they contribute to host defense by producing cytokines, chemokines and other inflammatory mediators. Apoptosis of epithelial cells may provide important defense mechanisms against viral infection, while excessive remodeling due to environmental injury-induced apoptosis is associated with chronic airway disease [13]. Thus, the interplay between epithelial cells and RSV infection involves a dynamic process whose course of progression and persistence depends upon how efficiently RSV is cleared by the epithelial cells. Primary differentiated normal human bronchial epithelial cells have, therefore, been considered a good model for *in vitro* analysis of the lung tissue response to respiratory virus infection and virus-host interactions. However, primary bronchial epithelial cells are expensive, difficult to culture, and variable depending on the patient source [14]. Furthermore, persistent RSV infections have been established in several human and animal cell lines of epithelial or immune origin [11], but it is unknown whether human bronchial epithelial cells permit viral persistent infections *in vitro*. Therefore, in this study, we have used human bronchial epithelial (16HBE) cells—a transformed human bronchial epithelial cell line—as an alternate approach for examining the molecular mechanisms of the *in vitro* progression of RSV infection under conditions that allow infection to occur over four generations. Dynamic analyses were used to investigate the function of bronchial epithelial cell inflammatory and matrix adherence molecules during this transition to further the understanding of the development of airway dysfunction following acute RSV disease outbreak.

## 2. Results and Discussion

### 2.1. *In Vitro* Model of Human Bronchial Epithelial (16HBE) Cells with RSV Infection over Four Generations

**Figure 1:**
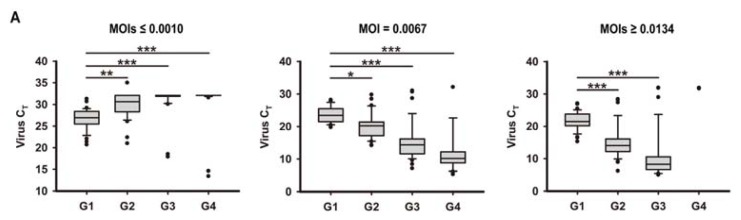

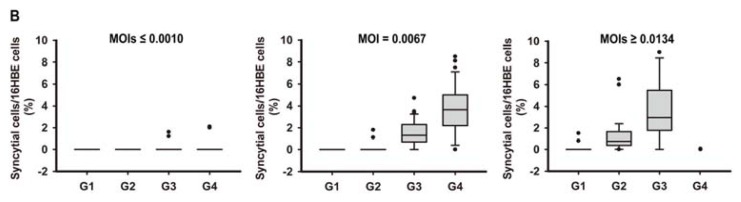
Effects of *multiplicity of infection (MOI)* on respiratory syncytial virus (RSV) RNA expression and the amount of syncytial cells in surviving human bronchial epithelial (16HBE) cells during successive passages. The box for each generation represents the interquartile range (25th–75th percentile) and the line within this box is the median value. Bottom and top bars of the whisker indicate the 10th and 90th percentiles, respectively. Outlier values are indicated (dot). (**A**) RSV RNA levels; and (**B**) the amount of syncytial cells in surviving RSV-infected 16HBE cells (*MOIs* ≤ 0.0010, *MOI* = 0.0067 or *MOIs* ≥ 0.0134) from the first generation (G1) to the fourth generation (G4). Symbols signify statistical significance, where *p < 0.05, **p < 0.01, and ***p < 0.001 compared with G1.

When RSV at *multiplicity of infections* (*MOIs*) ≤ 0.0010 was used, samples were tested clearly positive for RSV in the first generation (G1), but lost their positivity in G2 to G4 in most cases (Figure 1A, first panel). Absence of positivity remained up to the twentieth generation (data not shown). Very little apparent changes in cell morphologic characteristics was observed (Figure 1B, first panel), suggesting that viral clearance was effective for this low *MOI*. However, when RSV at *MOI* = 0.0067 was used to infect 16HBE cells, RSV was progressively cleared by 16HBE cells (or the 16HBE cells were destroyed) in G2 or G3 cells during successive passages. In most cases (about 80%), the 16HBE cells survived to G4 (Figure 1A, second panel). Surviving 16HBE cells in G2 showed similar healthy cell monolayer morphology as cultures of uninfected cells, while in G3 some small syncytia and irregularly shaped cells began to form, and in G4 large syncytia were observed with decreased numbers of cells (Figure 1B, second panel). G5 cultures were found to contain predominantly lysed cells, large amounts of syncytia, and a residue of scattered islands of cells adherent to the substratum that died shortly afterwards (data not shown). Though effective at promoting syncytia formation at G3, higher *MOIs* of RSV infection (*MOIs* ≥ 0.0134) led to minimal levels of survival (Figure 1A,B, third panels). Therefore, *MOI* 0.0067 was used to analyze progressive changes in inflammatory markers and matrix adherence at G1 to G4.

### 2.2. Dynamic Changes of Leukocyte Adherence to 16HBE Cells with Progressive RSV Infection Involve Intercellular Adhesion Molecule-1 (ICAM-1)

**Figure 2:**
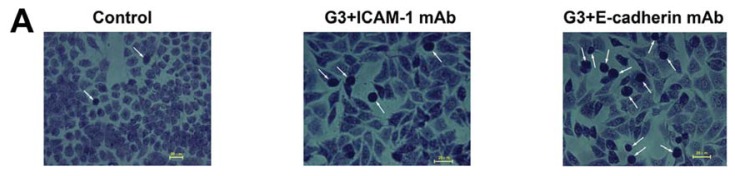

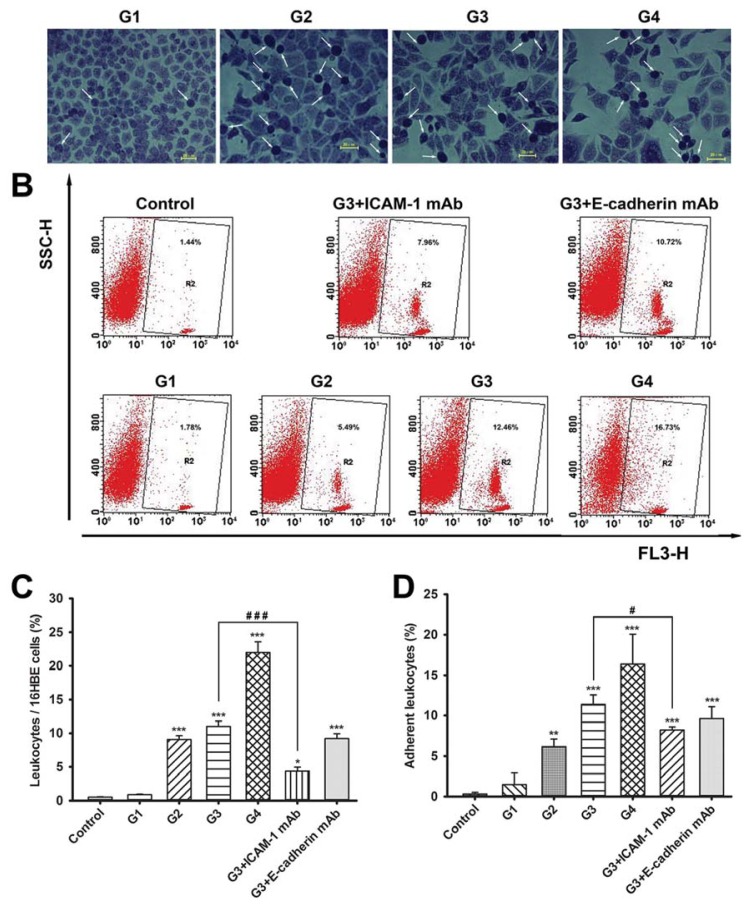
RSV-induced leukocyte adherence of 16HBE cells progressively increases over the course of infection and is dependent on intercellular adhesion molecule-1 (ICAM-1), but not E-cadherin. (**A**) Microphotograph showing the adhesion of leukocytes (white arrows) to 16HBE cells analyzed by Wright-Giemsa staining (200× magnification); (**B**) Representative plots are shown for leukocyte adherence analyzed using flow cytometry; (**C**) Histograms represent the mean (±SEM) number of adherent leukocytes analyzed by Wright-Giemsa staining; and (**D**) Histograms represent the number of adherent leukocytes analyzed using flow cytometry. Symbols signify statistical significance, where *p < 0.05, **p < 0.01, and ***p < 0.001 compared with control (non-inoculated cells); ^#^*p* < 0.05 and ^###^*p* < 0.001 compared with third generation (G3).

We used two different approaches to measure 16HBE cell inflammatory adherence. First, the number of leukocytes adhering firmly to the 16HBE cells was assessed using Wright-Giemsa staining at each generation. Representative images are shown in Figure 2A and results are quantified in Figure 2C. We found that the adherence of leukocytes to 16HBE cells was low in control cells. After infection with RSV, leukocyte adherence remained low in G1, but significantly and gradually increased 17.8- to 43.0-fold in G2 to G4 (*p* < 0.001 compared to control). To determine which adhesive molecules may be involved in leukocyte adhesion, we subjected cells in G3 to neutralizing antibodies against ICAM-1 and E-cadherin. Neutralization of ICAM-1 but not E-cadherin resulted in significant inhibition of leukocyte adherence to 16HBE cells compared with the G3 group.

Parallel experiments using fluorescence-activated cell sorting analysis yielded similar results (Figure 2B,D). There was no significant difference between the G1 and control cells. However, G2 to G4 16HBE cells showed progressively enhanced binding to leukocytes. Furthermore, leukocyte adherence was verified to be blocked by antibody to ICAM-1, but not E-cadherin.

### 2.3. Dynamic Changes of Extracellular Matrix (ECM) Adherence to 16HBE Cells with Progressive RSV Infection Involve E-cadherin

**Figure 3:**
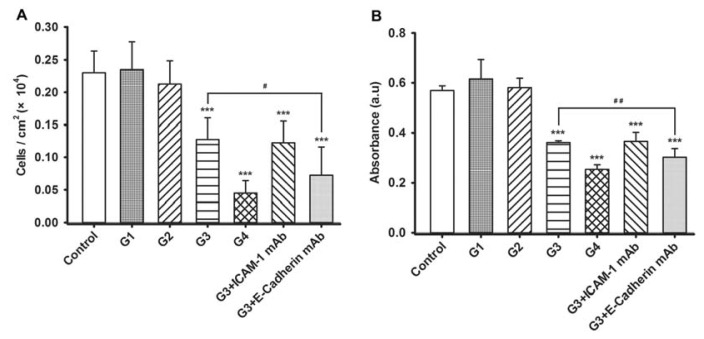
RSV-induced extracellular matrix (ECM) adherence of 16HBE cells progressively declines over the course of infection and is dependent on E-cadherin, but not ICAM-1. The adherent 16HBE cells to rat tail tendon collagen type I-coated plates was counted microscopically (**A**) or determined using crystal violet staining (**B**). Symbols signify statistical significance, where *p < 0.05, **p < 0.01, and ***p < 0.001 compared with control (non-inoculated cells); ^#^*p* < 0.05 and ^##^*p* < 0.01 compared with the third generation (G3). G3 + ICAM-1 mAb group, G3 cells in which ICAM-1 was blocked; G3 + E-cadherin mAb, G3 cells in which E-cadherin was blocked.

Next, to assess the effects of RSV infection on adherence of 16HBE cells to ECM, we coated plates with rat tail tendon collagen type I prior to plating cells and assessed numbers of adherent cells after infection. Results showed that similar numbers of 16HBE cells remained adherent in G1 and G2, while progressively fewer cells adhered to ECM in G3 and G4 (Figure 3A). Furthermore, in contrast to the results from the leukocyte adhesion assay, the adherence of 16HBE cells to ECM was significantly reduced by anti-E-cadherin antibody, but not ICAM-1 antibody. Results were verified by crystal violet staining, which yielded a reduced absorbance value in G3 and G4 compared to that of the control cells and verified the selective neutralization by E-cadherin antibody (Figure 3B).

### 2.4. Dynamic Changes of Cytokine and Chemokine Secretions by 16HBE Cells with Progressive RSV Infection and the Effects of ICAM-1 and E-cadherin Neutralizing mAbs

**Figure 4:**
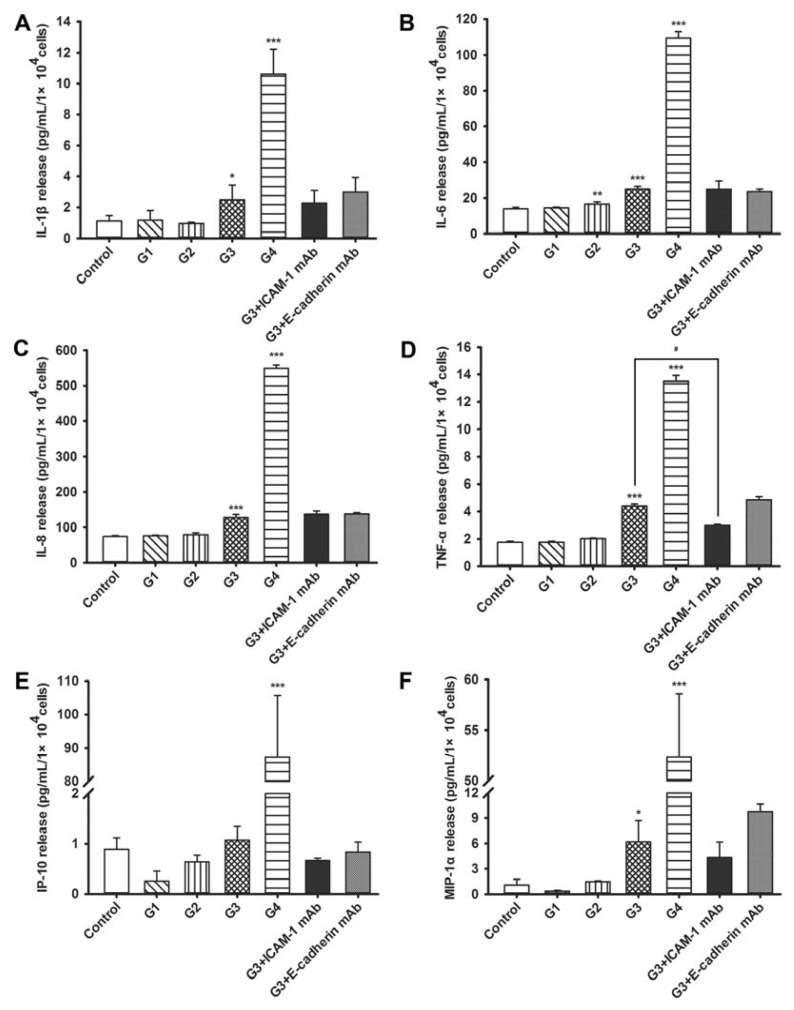
RSV-induced cytokine and chemokine secretion from 16HBE cells and the effect of ICAM-1 and E-cadherin neutralizing antibodies. The culture supernatants from the first to fourth generations (G1–G4) of RSV infection of 16HBE cells were analyzed for (**A**) interleukin (IL)-1β; (**B**) IL-6; (**C**) IL-8; (**D**) tumor necrosis factor-α (TNF-α); (**E**) interferon-γ-induced protein-10 (IP-10); and (**F**) macrophage inflammatory protein-1α (MIP-1α) concentrations using enzyme-linked immunosorbent assay (ELISA). Symbols signify statistical significance, where *p < 0.05, **p < 0.01, and ***p < 0.001 compared with control (non-inoculated cells) and ^#^*p* < 0.05 compared with the third generation (G3). G3 + ICAM-1 mAb group, G3 cells in which ICAM-1 was blocked; G3 + E-cadherin mAb, G3 cells in which E-cadherin was blocked.

In the current study, it was found that RSV infection up-regulated the release of an array of inflammatory molecules in 16HBE cells. As shown in Figure 4, IL-6 levels progressively increased in G2 through G4; interleukin (IL)-1β, IL-8, tumor necrosis factor-α (TNF-α), and macrophage inflammatory protein-1α (MIP-1α) increased in G3 and G4, and interferon-γ-induced protein-10 (IP-10) increased only in G4. ICAM-1 neutralization resulted in significantly decreased TNF-α levels compared with G3 group, while the levels of other cytokines and chemokines remained unchanged upon pre-incubation for 12 h with either ICAM-1-neutralizing antibody or E-cadherin-neutralizing antibody.

### 2.5. Dynamic Changes of Adhesive Molecule Expression or Secretion in 16HBE Cells with Progressive RSV Infection

**Figure 5:**
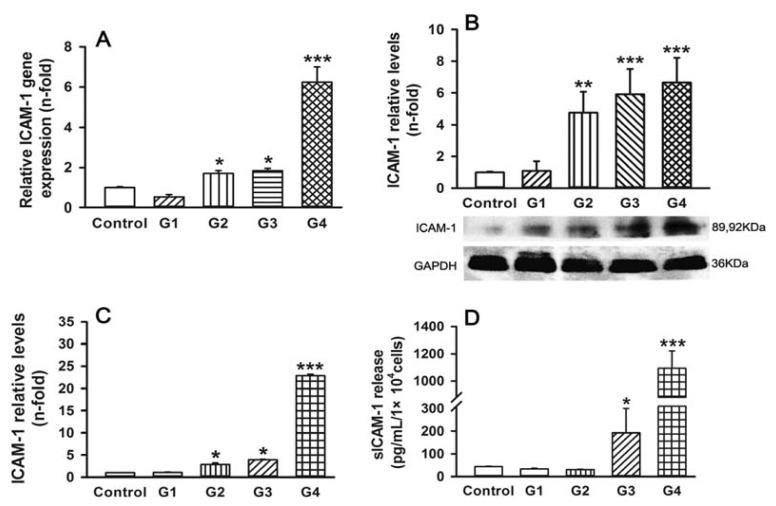
RSV-induced ICAM-1 expression and secretion in 16HBE cells. (**A**) Total RNA was analyzed for ICAM-1 mRNA levels; (**B**) Whole-cell lysates were analyzed for ICAM-1 protein levels using western blotting; (**C**) ICAM-1 protein levels were expressed in terms of absorbance at 450 nm using a whole-cell ELISA; and (**D**) Culture supernatants were analyzed for soluble ICAM-1 (sICAM-1) concentrations using ELISA. Symbols signify statistical significance, where *p < 0.05, **p < 0.01, and ***p < 0.001 compared with control (non-inoculated cells).

To further examine the role of adhesion molecules in the progression of RSV infection, we examined expression and secretion of ICAM-1 and E-cadherin throughout the successive generations of infection. As expected, with the exception of G1, the levels of ICAM-1 mRNA in RSV-infected cells gradually increased with each progressive generation and peaked in G4 (about 6.2-fold increase compared to control) (Figure 5A). Western blot analysis verified that ICAM-1 protein was significantly upregulated in G2 (about 4.8-fold), G3 (about 5.9-fold), and G4 (about 6.7-fold) compared with the control (Figure 5B). Similar results were obtained using either a whole-cell enzyme-linked immunosorbent assay (ELISA) assay for ICAM-1 expression (Figure 5C) or an ELISA of sICAM-1 in conditioned medium (Figure 5D; 4.4-fold increase in G3 and 25.2-fold increase in G4).

**Figure 6:**
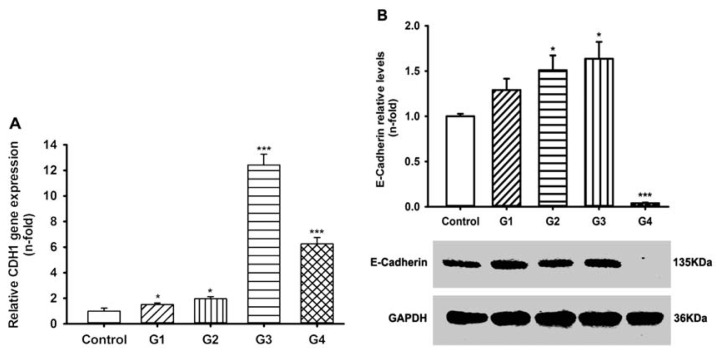
RSV-induced E-cadherin expression in 16HBE cells. (**A**) Total RNA was analyzed for *CADHERIN 1 (CDH1)* mRNA levels; and (**B**) Whole-cell lysates were analyzed for E-cadherin protein levels using western blotting. Symbols signify statistical significance, where *p < 0.05, **p < 0.01, and ***p < 0.001 compared with control (non-inoculated cells).

We also assessed the expression of the *CADHERIN 1 (CDH1)*, the gene for E-cadherin. Real-time polymerase chain reaction (PCR) results showed that *CDH1* mRNA expression in RSV-infected 16HBE cells was upregulated compared to control until G4. However, it was dramatically decreased in G4 relative to G3 (Figure 6A). Western blot assay results supported this finding: while E-cadherin protein expression was increased in G3 relative to the control, it was almost undetectable in G4 (Figure 6B). These finding suggest that both ICAM-1 and E-cadherin are increased in progressive generations of RSV infection up to G3, but that E-cadherin expression is dramatically reduced in G4.

### 2.6. Discussion

A number of studies including our previous studies have confirmed that RSV mRNA existed in mice lung tissue homogenate at least 100 days after RSV infection, and airway epithelial cells are the potential sites of RSV persistence. Persistent RSV infections have been established in several human and animal epithelial cell lines [11]; however, it has been controversial whether human epithelial cells of bronchial origin can permit viral persistent infections *in vitro*. We sought to determine whether RSV can infect the human bronchial epithelial cell line—16HBE cells—over multiple generations. The results showed that when RSV at a *multiplicity of infection* (*MOI*) = 0.0067 was used to infect 16HBE cells, RSV survived to G4. The establishment of an *in vitro* model for infection of human bronchial epithelial cells that persists for four generations prior to death provides a novel system for characterizing persistent RSV mechanisms.

RSV-induced airway dysfunction is likely the result of direct damage induced by viral replication but may also be facilitated by inflammation. Previous experimental evidence has suggested that an excessive inflammatory response triggered by the host plays a major role in the development of clinical manifestation of RSV infection [15]. *In vivo*, cytokines/chemokines secreted from infected cells can regulate the recruitment of neutrophils, mononuclear cells, or lymphocytes to the site of RSV infection and can stimulate leukocyte degranulation or inflammatory mediator release [16]. RSV-infected airway epithelial cells secrete high levels of neutrophil-recruiting chemoattractant IL-8, as well as other proinflammatory cytokines [17]. Therefore, we measured the secreted levels of a panel of cytokines and chemokines using ELISA. Consistent with previous *in vivo* studies of RSV infection [18–21], these results indicate that cytokine and chemokine induction upon RSV infection progressively increases and peaks at G4, and that the induction of TNF-α may be dependent on ICAM-1.These data are consistent with previous studies suggesting that the adherence of 16HBE cells to leukocytes is primarily mediated by increased ICAM-1 expression upon RSV infection [22]. ICAM-1 is also known to facilitate RSV entry and infection of human epithelial cells by binding to its F protein, which is important for viral replication and infection [19], and therefore, this increase might serve both to exacerbate adherence and to enhance progressive infectivity.

E-cadherin is normally expressed in epithelial cells, and the dissociation of the E-cadherin-mediated adherens’ junctions is related to a loss of epithelial integrity [23,24]. Additionally, β-catenin, which can bind to the cytoplasmic tail of E-cadherin, serves multiple roles in cell architecture maintenance [25]. Thus, the dramatic loss of E-cadherin in G4 could explain the decrease in the ability of 16HBE cells to adhere to ECM in G4 and may also play a key role in RSV-induced bronchial epithelial cell shedding. RSV-positive illnesses are associated with increased epithelial shedding as a viral mechanism of promoting infection [26].

These observations above demonstrate a complex array of events that are set into motion in RSV infected 16HBE cells, which results in inflammatory mobilization and epithelial shedding, and eventually leads to cell death. Viral persistence in the lungs is generally caused by interferon, viral mutations, or changes in both virus and host cells and more than one factor is usually responsible [27]. The success of RSV in establishing productive infections in human airway epithelia depends on viral expression mechanisms for the evasion of innate and acquired immune responses. The nonstructural (NS) proteins of RSV, NS1, and NS2 inhibit interferon (IFN)-α and IFN-β production by decreasing the proinflammatory transcription factors interferon regulatory factor (IRF)-3, nuclear factor-κB (NF-κB), and signal transducer and activator of transcription protein (STAT)-2 [28,29]. On the other hand, RSV clearance also requires the induction of a balanced T helper (Th)1/Th2 adaptive immune response that promotes the production of neutralizing antibodies [preferably mucosal immunoglobulin A (IgA)] and the induction of IFN-ã secreting cytotoxic CD8^+^ T cells [8]. In the 16HBE *in vitro* culture system used in this study, no other immune cells were available to participate in an adaptive immune response for clearing virus. Moreover, subgroup A strains may yield significantly higher viral loads than subgroup B strains and cause more severe clinical syndromes [30]. The passage of the cells might also have further facilitated the viral spread by disrupting both cell-to-cell contact and the innate cellular response that may protect neighboring cells. Therefore, achieving a balance between RSV strain A2 persistence and 16HBE cell survival posed a formidable challenge. However, the experimental conditions that we established produced a four-generation model that allowed us to monitor the molecular mechanisms of RSV infection over its course of progression before succumbing to the virus.

Inflammation is an essential component of the host defense; however, an overly vigorous response against microbes may be deleterious to the host and lead to impaired organ function [31]. In our model, the exacerbated RSV-triggered immune-inflammatory response may be the underlying cause of rapid death of G5 16HBE cells. Our results using neutralizing antibodies indicate that TNF-α secretion may depend on ICAM-1. However, some studies showed adhesive molecule expression can be induced by cytokines such as TNF-α, interferon-γ and IL-1 in inflammatory situations [32–34]. Therefore, cross-talk between TNF-α secretion and ICAM-1 expression may be involved in triggering an inflammatory cascade towards exacerbated inflammation. The induction of ICAM-1 in 16HBE cells upon RSV infection is unlikely to be solely induced by these cytokines, because its induction precedes that of the cytokines. Respiratory epithelium expresses Toll-like receptors (TLRs) and Retinoic acid-inducible gene-I (RIG-I)-like receptors (RLRs) that sense viral RNA [31]. After attaching to epithelial cells, RSV induces the transcription of the genes promoting an anti-viral response, such as cytokines and chemokines, through the activation of a subset of transcription factors, including NF-κB and activator protein-1 (AP-1) [35,36]. The elucidation of transcriptional mechanisms that lead to the activation of these inflammatory mediators may help to further our understanding of cellular responses to RSV.

## 3. Experimental Section

### 3.1. RSV Preparation

The RSV strain A2 and the HEp-2 cell line were kindly provided by Professor Rong Zhou (Guangzhou Children’s Hospital, Guangzhou, China; stored in their lab). The RSV was expanded in HEp-2 monolayer cultures. HEp-2 cells were maintained in Opti-Mem I medium (Invitrogen, Carlsbad, CA, USA) supplemented with 5% fetal bovine serum (FBS) supplied by Gibico BRL (Ground Island, NY, USA) and 1% penicillin-streptomycin. HEp-2 cells (60%–70% confluent) were infected with RSV at *MOI* of 0.5 for 1.5 h at 37 °C, washed two times, and then incubated at 37 °C. The infection was allowed to proceed until syncytia were observed. The cells were subjected to three successive freeze-thaw cycles followed by resuspension in fresh medium. The RSV was further purified with two rounds of centrifugation at 2500× *g* for 10 min at 4 °C, filtered through a 0.22-μm filter, and aliquoted and stored at −80 °C until use.

### 3.2. 16HBE Cell Culture and RSV Infection

16HBE cells were cultured in Dulbecco’s Modified Eagle’s Medium (DMEM) supplied by Gibico BRL (Ground Island, NY, USA) supplemented with 10% FBS at 37 °C under 5% CO_2_ in humidified air. 60%–70% confluent monolayer cultures of 16HBE were infected with RSV at *MOI* of 0.00025, 0.0005, 0.0010, 0.0067, 0.0134, 0.0268 and 0.0536 according to experiments in advance. The virus was allowed to incubate for 2 h at 37 °C in serum-free DMEM. Thereafter, non-absorbed virus was removed and washed two times, then the cells (the zero generation, G0) were cultured in fresh medium with 2% FBS and subcultured as needed. Cells were passaged two times per week. Each *MOI* experiment was repeated 30 times. 16HBE cells non-inoculated with RSV were used as control.

### 3.3. Assessment of RSV Infection by Real-Time PCR Analysis and Counting the Syncytial Cells

During passages, persistence was verified and monitored using a Respiratory Syncytial Virus Real-time PCR Kit (Guangzhou Huayin Medicine Biotechnology Co. Ltd., Guangzhou, China). This TaqMan^®^-based real-time PCR kit includes the reagents for nucleic acid extraction, real-time PCR, high positive, and negative control materials. According to the instructions, the samples were considered negative for RSV when the threshold cycle number (*C*_T_) > 32.0. Conservatively, samples with a *C*_T_ value ≤ 28.9 were considered clearly positive. All samples including the no-template control were tested in triplicate to ensure repeatability. The number of syncytial cells in each generation was analyzed using a Leica DC200 digital camera system (Leica Microsystems, Wetzlar, Germany). For mock infection, uninfected 16HBE cell culture monolayers were harvested and treated as described above. Cells infected with RSV (*MOI* = 0.0067) from generation G1 to G4 were used to analyze inflammatory and matrix adherence only when the cells of G5 were dead.

In antibody blocking experiments, near-confluent G3 16HBE cells were pre-incubated with vehicle (DMEM), mouse anti-intercellular adhesion molecule-1 (ICAM-1) (Anti-human, Klon LB-2) monoclonal antibody (10 μg/mL; Santa Cruz Biotechnology, Santa Cruz, CA, USA), or rabbit anti-E-cadherin polyclonal antibody (1:100; Cell Signaling Technology Inc., Beverly, MA, USA) for 12 h in DMEM (37 °C, 5% CO_2_). In addition, the comparison was with non-specific antibody on the G3 cells. Each experiment was repeated at least three times.

### 3.4. Isolation of Peripheral Blood Leukocytes

Human fresh blood was drawn from healthy donors into heparin-coated vacuum tubes and leukocytes were prepared in a laminar flow cabinet to minimize exposure to endotoxin as described previously [37] with slight modifications. Briefly, whole blood was diluted 1:1 with 0.5 N Hanks’ buffered salt solution (HBSS). After centrifugation at 100× *g* for 10 min, the supernatant containing the leukocytes was collected and layered on Lymphoprep (density 1.077 g/mL; Nycomed Pharma, Oslo, Norway). Following subsequent centrifugation at 800× *g* for 25 min, the leukocyte layer at the interface was collected and washed three times with HBSS. The cells were resuspended in complete medium at a final density of 1 × 10^7^ cells/mL and used in the adhesion assay within 1 h of isolation. Leukocytes isolated in this manner were viable (>99%) as determined by trypan blue exclusion.

### 3.5. Leukocyte-16HBE Cell Adherence Assay

Leukocyte-16HBE cell adherence was analyzed using Wright-Giemsa staining. 16HBE cells in 24-well plates were washed twice with phosphate-buffered saline (PBS). The leukocytes (5 × 10^4^ cells/well) were then added to the 16HBE cell cultures to a total volume of 0.4 mL and incubated for 12 h at 37 °C. After two washes with PBS, cells were stained with Wright-Giemsa solution for 15 min, rinsed with distilled water, and air dried. Four randomly chosen fields per well were analyzed, and the number of adherent leukocytes was determined using a Leica DC200 digital camera system. Adherence was expressed as a percentage of the ratio of leukocytes binding to 16HBE cells.

For verification of results, leukocyte-16HBE cell adherence was also assayed by flow cytometer using a modification of the method of Floreani *et al.* [38]. 16HBE cells in six-well plates were washed twice with PBS. The leukocytes (2 × 10^5^ cells/well) were then added to a total volume of 2.0 mL and incubated for 12 h at 37 °C. Non-adherent cells were removed and the remaining cells were collected and incubated with anti-CD45 PerCP (BD Biosciences, San Jose, CA, USA) for 20 min at 25 °C. After washing, the cells were resuspended in 300 μL of PBS and analyzed on a FACSCalibur flow cytometer (Becton Dickinson, Mountain View, CA, USA) with data stored in list mode files. The percentage of leukocytes binding to 16HBE cells was evaluated first by live gating on 16HBE cells using forward and side scatter and then by a combination of anti-CD45-PerCP fluorescence and side scatter. Ten thousand events were measured in the gate and the data were analyzed using CELLQuest Pro software (Becton Dickinson, CA, USA).

### 3.6. 16HBE Cell-Extracellular Matrix (ECM) Component Adherence Assay

Six-well plates were coated with rat tail tendon collagen type I (Shengyou Biotechnology Co. Ltd., Hangzhou, China) according to the manufacturer’s instructions. Thereafter, 3.0 × 10^5^ 16HBE cells were added to each well for 2 h at 37 °C under 5% CO_2_ in humidified air. Non-adherent cells were removed and the remaining cells were harvested and counted under a light microscope. The mean cellular adhesion rate was calculated as adherent cells_coated well_ – adherent cells_background_. Plastic dishes served as the background control.

Assessment of ECM adherence of the 16HBE cells was also performed using 96-well culture plates coated with rat tail tendon collagen type I. 16HBE cells (1.0 × 10^4^) were added to each well for 2 h at 37 °C under 5% CO_2_ in humidified air. Non-adherent cells were removed and adherent cells were fixed in 4% (*v*/*v*) paraformaldehyde (Sigma-Aldrich, St. Louis, MO, USA) for 10 min, stained with 0.1% (*w*/*v*) crystal violet (Sigma-Aldrich) in 20% (*v*/*v*) methanol for 30 min, and then washed with distilled water. Adherent levels were quantified at 570 nm using a microplate reader (MK3; Thermo, Sunnyvale, CA, USA) after the addition of 50 μL of 0.2% (*v*/*v*) Triton X-100 (Sigma-Aldrich) in distilled water. Bovine serum albumin (BSA; Sigma-Aldrich)-coated and poly-l-lysine (Sigma-Aldrich)-coated wells served as the negative and positive controls, respectively.

### 3.7. Assessment of Gene Expression by Real-Time Reverse Transcription (RT)-PCR

Total RNA was isolated from control and RSV-infected 16HBE cells using TRIzol reagent (Invitrogen). cDNA was synthesized using a Primescript™ RT Reagent Kit (TaKaRa, Tokyo, Japan) with oligo (dT) primer. The sequences of the PCR primers used were: ICAM-1 (forward, 5′-AGCCAACCAATGTGCTATTCAAAC-3′; reverse, 5′-CACCTGGCAGCGTAGGGTAA-3′); E-cadherin (CDH1) (forward, 5′-TACACTGCCCAGGAGCCAGA-3′; reverse, 5′-TGGCACCAGTGTCCGGATTA-3′); glyceraldehyde 3-phosphate dehydrogenase (GAPDH) (forward, 5′-GCACCGTCAAGGCTGAGAAC-3′; reverse, 5′-TGGTGAAGACGCCAGTGGA-3′). Real-time PCR was performed using 2 μL of template DNA, 10 μL of FastStart Universal SYBR Green Master (ROX) (Roche, Mannheim, Germany), and 300 nM of forward and reverse primer in a final volume of 20 μL. PCR was carried out using an ABI 7500 Real-Time PCR system (Applied Biosystems, Carlsbad, CA, USA): 10 min of initiated activation at 95 °C and 40 cycles of amplification at 95 °C for 15 s and 60 °C for 60 s. Fluorescent products were detected in the last step of each cycle. Samples containing primer dimers were excluded by melting curve analysis and identification of the products by agarose gel electrophoresis. Data were recorded using the 7500 software V2.0 program (Applied Biosystems, Carlsbad, CA, USA). Relative expression levels were calculated using the 2^−ΔΔ^*^C^*^T^ method, with Δ*C*_T_ = C_T(Target gene)_ − C_T(GAPDH)_ and ΔΔ*C*_T_ = Δ*C*_T(Treatment)_ − Δ*C*_T(Control)_. Experiments were repeated three times.

### 3.8. Western Blot Analysis

Control and RSV-infected 16HBE cells were lysed in protease inhibitor cocktail solution (Roche, Indianapolis, IN, USA). Cell lysates (70 μg) were separated on 8%–10% sodium dodecyl sulfate-polyacrylamide gels (Bio-Rad, Hercules, CA, USA) and then transferred onto polyvinylidene fluoride membrane (Millipore, Billerica, MA, USA). Membranes were blocked with 5% skim milk and incubated with rabbit anti-CD54 antibody and E-cadherin antibody (Cell Signaling Technology Inc.) at 4 °C overnight. After being washed, membranes were incubated with horseradish peroxidase (HRP)-conjugated goat anti-rabbit IgG (1:5000; ProteinTech Group Inc., Chicago, IL, USA) for 1 h at room temperature. Antibody-antigen complexes were then detected using an ECL chemiluminescent detection system (Gene Co., Ltd., Hong Kong, China). GAPDH (Bioworld Technology Inc., Minneapolis, MN, USA) was used as a loading control. A densitometry analysis was performed using AlphaEase software version 2200 (2.2 d) (Alpha Innotech Co., San Leandro, CA, USA).

### 3.9. Enzyme-Linked Immunosorbent Assay (ELISA)

ICAM-1 expression in 16HBE cells was also measured using a semi-quantitative whole-cell ELISA as previously described [39]. Concentrations of interleukin (IL)-1β, IL-6, IL-8, tumor necrosis factor-α (TNF-α; R&D Systems, Minneapolis, MN, USA), soluble ICAM-1 (sICAM-1; Invitrogen), interferon-γ-induced protein-10 (IP-10; Invitrogen), and macrophage inflammatory protein-1α (MIP-1α; Invitrogen) in the culture supernatant were determined using ELISA kits according to the manufacturer’s protocol. Absorbances were measured using a microplate reader.

### 3.10. Statistical Analysis

Data are presented as mean (±SEM) and were graphed using Sigma Plot 12.0 (Systat Software, Inc., San Jose, CA, USA). Differences between groups were assessed by one-way analysis of variance analysis. All analyses were performed using Statistical Package for the Social Sciences (SPSS) statistical software for Windows version 11.5 (SPSS, Inc., Chicago, IL, USA). A value of *p* < 0.05 was considered statistically significant.

### 3.11. Ethics Statement

Human peripheral blood samples were collected from healthy adult donors. The study protocol was approved by the ethics committee of Xiangya Hospital, Central South University, and written informed consent from all participants involved in our study was obtained before the study commenced.

## 4. Conclusions

These data demonstrate the susceptibility of 16HBE cells to RSV and their capacity to produce long-term progressive RSV infection, which may contribute to inflammation mobilization and epithelial shedding. Understanding of the progression of RSV infection may provide new insight into the cellular mechanisms of RSV, and therefore, suggest possible new strategies for treatment.
